# The cardiovascular-kidney-metabolic syndrome in 2025: a year in review

**DOI:** 10.1590/2175-8239-JBN-2026-0052en

**Published:** 2026-11-09

**Authors:** Rogerio Passos, Ana Flávia Moura, Thyago Proença de Moraes, Felipe Costa Neves, Luis Claudio Santos Pinto, Fabiano Bichuette Custodio, José A. Moura-Neto, Roberto Pecoits-Filho

**Affiliations:** 1 Sociedade Brasileira de Nefrologia, São Paulo, SP, Brazil.; 2 DaVita Brasil, São Paulo, SP, Brazil.; 3 Hospital Israelita Albert Einstein, São Paulo, SP, Brazil.; 4 Escola Bahiana de Medicina e Saúde Pública, Salvador, BA, Brazil.; 5 Pontifícia Universidade Católica do Paraná, Escola de Medicina, Curitiba, PR, Brazil.; 6 Hospital Ana Nery, Salvador, BA, Brazil.; 7 Universidade Federal do Pará, Belém, PA, Brazil.; 8 Universidade Federal do Triângulo Mineiro, Uberaba, MG, Brazil.; 9 University of Michigan, Division of Nephrology, Ann Arbor, United States.; 10 Arbor Research Collaborative for Health, ClinEpi Program, Ann Arbor, United States.

**Keywords:** Metabolic Syndrome; Renal Insufficiency, Chronic, Health Plan Implementation, Heart Disease Risk Factors, Metabolic Diseases

## Abstract

**Introduction:**

The cardiovascular-kidney-metabolic (CKM) syndrome has emerged as an integrated clinical framework reflecting shared pathobiology and overlapping risk trajectories across cardiovascular, renal, and metabolic diseases. In 2025, this paradigm advanced from a conceptual construct toward an operational strategy, informed by randomized clinical trials, mechanistic studies, and implementation-focused analyses.

**Main findings:**

This Year in Review synthesizes the major randomized clinical trials with prespecified analyses, secondary analyses, mechanistic studies, and trial design reports published during 2025 shaping contemporary CKM care. Evidence consolidated sodium-glucose cotransporter-2 inhibitors and nonsteroidal mineralocorticoid receptor antagonists as foundational therapies across heart failure (HF) and chronic kidney disease (CKD) phenotypes, with expanding data on earlier initiation, combination strategies, and phenotype-guided application. Incretin-based therapies addressed obesity-driven CKM phenotypes, particularly HF with preserved ejection fraction, while mechanistic trials and biomarker analyses strengthened biological plausibility across inflammatory, endothelial, metabolic, and fibrotic pathways. Advances in vascular prevention highlighted the relevance of residual inflammatory and atherosclerotic risk beyond lipid lowering, and studies targeting anemia, congestion, and acute kidney injury expanded the operational scope of CKM management. Parallel developments in risk stratification incorporated frailty, adiposity, biomarker trajectories, and machine-learning tools, supporting a shift away from single-organ severity thresholds toward integrated, trajectory-based care.

**Conclusions:**

The 2025 evidence base reinforces CKM syndrome as a systems-level disease state addressed through earlier intervention, rational pathway layering, and integrated care models. Future priorities include definitive outcomes trials of combination strategies, pragmatic implementation studies, and improved inclusion of advanced CKD and dialysis populations to ensure equitable translation of CKM advances into clinical practice.

## INTRODUCTION

Across 2025, the cardiovascular-kidney-metabolic (CKM) syndrome progressed from a descriptive label for clustered comorbidity to a more actionable clinical framework with direct implications for prevention, screening, phenotype definition, risk stratification, and therapeutic sequencing^1^. Importantly, this evolution reflects a shift toward a holistic, multi-organ construct, rather than disease-specific silos, positioning CKM as a *continuum* that spans cardiovascular, renal, and metabolic domains.

Three convergent themes were repeatedly reinforced. First, heart failure (HF), atherosclerotic cardiovascular disease, chronic kidney disease (CKD), and dysglycemia/obesity share overlapping pathobiology—systemic inflammation, endothelial dysfunction, maladaptive fibrotic signaling, and altered substrate utilization—that plausibly drives parallel trajectories of organ injury and clinical instability^2^. Second, earlier initiation and rational layering of disease-modifying therapies, together with comprehensive lifestyle modifications, are increasingly required to mitigate the residual risk that persists when care remains anchored to single-organ severity, late-stage disease, or reactive treatment paradigms^3^. Third, CKM risk assessment is moving beyond estimated glomerular filtration rate (eGFR) or ejection fraction (EF) thresholds toward multidimensional phenotyping, integrating biomarkers, frailty, adiposity-related metrics, and machine-learning approaches to better align therapeutic intensity with biologic risk^4^. Collectively, these themes underscore the growing need for integrated, longitudinal care models that coordinate prevention and treatment strategies across specialties and disease stages, rather than fragmented organ-specific management.

The growing clinical relevance of CKM syndrome extends beyond its pathophysiological complexity and is increasingly reflected in its population-level burden. CKM syndrome accounted for more than 3.3 million deaths in the United States between 2010 and 2019, with mortality increasing steadily over the decade and marked disparities according to age, race, and geographic region^5^. Similarly, a nationwide population-based study from China demonstrated the high prevalence of CKM syndrome and its close association with adverse clinical outcomes, highlighting that this emerging construct represents a global health challenge extending well beyond Western populations^6^. Complementing these observations, prospective data from the UK Biobank demonstrated a graded increase in all-cause, cardiovascular, cancer, and other-cause mortality across advancing CKM stages while also showing that adherence to a healthy lifestyle substantially attenuated these risks irrespective of disease stage^7^. Together, these findings reinforce the growing population-level impact of CKM syndrome and underscore the need for integrated prevention strategies, early intervention, and continuous appraisal of emerging therapeutic advances.

The year’s trials and prespecified analyses further consolidated evidence for sodium-glucose cotransporter-2 (SGLT2) inhibitors^8,9^, nonsteroidal mineralocorticoid receptor antagonism (MRA) with finerenone^10,11^, and incretin-based strategies in obesity-related HF phenotypes^12,13,14,15^. In parallel, emerging programs targeting anemia^16,17,18,19,20^, congestion and hemodynamic management^21,22,23,24,25^, and albuminuria reduction as a therapeutic endpoint^26^ expanded the operational scope of CKM care. In this Year in Review, we adopt a broad CKM perspective to synthesize the major randomized clinical trials published in 2025, emphasizing how integrated, pathway-based interventions inform contemporary prevention and management across the cardiovascular-renal-metabolic spectrum, rather than prioritizing a single-organ lens. Table 1 summarizes the major clinical studies published during 2025, including landmark randomized clinical trials together with influential prespecified analyses, mechanistic studies, and trial design reports that shaped the evolving cardiovascular-kidney-metabolic framework.

**Table 1:** Landmark randomized clinical trials and key analyses published during 2025 that shaped the cardiovascular-kidney-metabolic (CKM) syndrome framework.

Study	2025 publication type	*N*	Population	Intervention	Primary endpoint	Main findings	Clinical relevance
SOUL^8^	Prespecified analysis	9,650	Adults ≥50 years with T2D and established ASCVD and/or CKD	Oral semaglutide vs. placebo	Major adverse cardiovascular events (MACE)	Consistent reduction in MACE irrespective of background SGLT2 inhibitor therapy	Confirms cardiovascular benefit of oral GLP-1 receptor agonist therapy
SUMMIT^12^	Primary randomized clinical trial	731	HFpEF and obesity	Tirzepatide	Cardiovascular death or worsening heart failure	Reduced composite cardiovascular outcome and improved health status	Establishes obesity-directed therapy as a strategy for HFpEF
FLOW^103^	Prespecified analysis	3,533	T2D and CKD	Semaglutide	Kidney and cardiovascular outcomes	Consistent kidney and cardiovascular protection across clinically relevant subgroups	Reinforces GLP-1 receptor agonists as kidney-protective therapy
DECODE-CKD^104^	Mechanistic randomized clinical trial	222	CKD	Dapagliflozin	Change in left ventricular mass index	Significant reduction in left ventricular mass index	Provides mechanistic evidence supporting cardiac reverse remodeling
MIRO-CKD^17^	Trial design and baseline report	324	CKD with albuminuria	Balcinrenone + dapagliflozin	Change in UACR	Study rationale and baseline characteristics reported	Evaluates dual mineralocorticoid receptor and SGLT2 inhibition
CONFIDENCE^64^	Trial design and baseline report	807	T2D and CKD	Finerenone + empagliflozin	Change in UACR	Study rationale and baseline characteristics reported	Supports combination CKM pathway inhibition
DAPA ACT HF–TIMI 68^30^	Primary randomized clinical trial	2,401	Patients hospitalized with acute heart failure	Dapagliflozin	Cardiovascular death or worsening heart failure	Neutral primary endpoint; accompanying meta-analysis supported early in-hospital initiation	Supports early SGLT2 inhibitor initiation based on the totality of evidence
FINEARTS-HF^47^	Prespecified analyses	6,001	HFmrEF/HFpEF	Finerenone	Multiple prespecified cardiovascular and kidney outcomes	Expanded evidence across clinically relevant CKM phenotypes	Extends the role of nonsteroidal mineralocorticoid receptor antagonists across the CKM spectrum
FAIR-HF2^51^	Primary randomized clinical trial	1,105	HFrEF with iron deficiency	Intravenous ferric carboxymaltose	Cardiovascular death or heart failure hospitalization	Neutral primary endpoint with improvement in functional status	Supports individualized correction of iron deficiency
IRONMAN^55^	Secondary analyses	1,137	Heart failure with iron deficiency	Intravenous ferric derisomaltose	Age-stratified efficacy and safety outcomes	Consistent safety and efficacy across clinically relevant subgroups	Supports individualized intravenous iron therapy
SURPASS-CVOT^69^	Primary cardiovascular outcomes trial	13,299	T2D with established ASCVD	Tirzepatide	Major adverse cardiovascular events (MACE)	Demonstrated cardiovascular safety compared with dulaglutide	Reinforces the cardiovascular safety profile of tirzepatide
REDEFINE 1^72^	Primary randomized clinical trial	3,417	Obesity	CagriSema	Relative change in body weight (co-primary endpoint: ≥5% weight loss)	Superior weight reduction vs. placebo	Advances combination incretin therapy for obesity
REDEFINE 2^73^	Primary randomized clinical trial	1,206	T2D and obesity	CagriSema	Relative change in body weight	Superior weight reduction	Expands evidence for obesity management in T2D
ACHIEVE-1^62^	Primary randomized clinical trial	559	T2D	Oral orforglipron	HbA1c reduction	Superior glycemic control and weight reduction	Expands oral incretin-based therapeutic options

Abbreviations – ASCVD: atherosclerotic cardiovascular disease; CKD: chronic kidney disease; CKM: cardiovascular-kidney-metabolic; CV: cardiovascular; HF: heart failure; HFmrEF: heart failure with mildly reduced ejection fraction; HFpEF: heart failure with preserved ejection fraction; HFrEF: heart failure with reduced ejection fraction; MACE: major adverse cardiovascular events; SGLT2: sodium-glucose cotransporter-2; T2D: type 2 diabetes; UACR: urinary albumin-to-creatinine ratio; GLP-1: glucagon-like peptide-1; HbA1c: glycated hemoglobin.

Notes – The 2025 publication type column identifies the nature of the publication included in this review (primary randomized clinical trial, prespecified analysis, secondary analysis, mechanistic randomized study, or trial design/baseline report). For studies whose original trial results were published before 2025, only the analyses or reports published during 2025 that provided new clinically relevant insights were included. Clinical relevance summarizes the principal contribution of each publication to the evolving CKM framework.

Collectively, the 2025 evidence base strengthens a unifying narrative: CKM syndrome is increasingly managed as shared biology addressed through layered pathway modification, while phenotyping and implementation—how therapies are selected, started, and sustained—become as central as drug development itself^1,2,3,4^.

## METHODS

This Year in Review was conducted as a structured narrative review of the cardiovascular-kidney-metabolic (CKM) literature published between January 1 and December 31, 2025. Relevant studies were identified through searches of PubMed/MEDLINE and Embase, complemented by manual searches of major cardiovascular, nephrology, diabetes, and internal medicine journals. Articles published online ahead of print during 2025 were considered eligible when they represented the first public dissemination of the study findings.

Priority was given to randomized clinical trials evaluating clinically relevant outcomes. Prespecified analyses, secondary analyses, mechanistic randomized studies, and trial design or baseline reports published during the same period were also included when they provided important insights into the evolving CKM framework or had substantial implications for contemporary clinical practice. Narrative reviews, expert opinions, case reports, and studies without direct relevance to CKM syndrome were not systematically included.

## RESULTS AND DISCUSSION

### Mechanistic convergence: inflammation, endothelial dysfunction, mitochondrial dysfunction, fibrosis, and metabolism

A defining direction in 2025 research was the refinement of mechanistic hypotheses explaining why interventions developed for glycemic control or neurohormonal modulation produce clinically meaningful effects across HF and CKD beyond their canonical domains. Experimental and translational work proposed that early SGLT2 inhibition after myocardial infarction (MI) in people with diabetes intersects with innate immune activation—particularly the NLRP3 inflammasome—and senescence-associated secretory phenotype pathways, providing a coherent anti-inflammatory and anti-senescent framing within CKM biology^27^. In parallel, exploratory analyses from large HF trial populations suggested that inflammatory mediators, including interleukin-6, track with progression and outcomes, consistent with inflammation functioning both as a marker of risk and a plausible mediator of downstream organ injury^28,29^.

Endothelial dysfunction continued to emerge as a unifying substrate linking cardiometabolic comorbidity to HF across the EF spectrum. Multi-cohort analyses associated endothelial alterations with cardiac function and clinical outcomes from health through HF, aligning with the hypothesis that comorbidity-driven inflammation impairs microvascular function, amplifies congestion biology, and increases kidney vulnerability—particularly in HF with preserved EF (HFpEF) phenotypes^30^.

Complementary proteomic analyses in diabetes and HF populations underscored biologic heterogeneity across EF categories, supporting the view that pathobiology relevant to therapeutic response and residual risk does not map cleanly onto EF alone^31^.

An additional dimension of this evolving multisystem paradigm is the growing recognition of metabolic dysfunction-associated steatotic liver disease (MASLD) and metabolic dysfunction-associated steatohepatitis (MASH) as important amplifiers of CKM syndrome. Rather than representing an isolated hepatic disorder, MASLD/MASH is increasingly understood as part of a complex network of metabolic dysfunction characterized by insulin resistance, chronic low-grade inflammation, oxidative stress, and progressive fibrosis, all of which contribute to cardiovascular and kidney injury^32^. The severity of liver fibrosis has been independently associated with adverse cardiovascular and renal outcomes, suggesting that hepatic involvement may further identify patients at particularly high cardiometabolic risk. Importantly, recent randomized clinical trials have shown that therapies already established within the CKM framework also provide meaningful hepatic benefits. Dapagliflozin significantly improved histological features of MASH, including steatohepatitis resolution and fibrosis improvement^33^, while semaglutide demonstrated substantial improvements in both steatohepatitis resolution and liver fibrosis in patients with biopsy-confirmed MASH^34^. These findings reinforce the concept that targeting shared metabolic pathways may simultaneously improve hepatic, cardiovascular, and renal outcomes, further supporting an integrated approach to CKM management.

Taken together, 2025 data further anchored CKM syndrome as a systems-level disease state, in which cardiorenal outcomes reflect integrated inflammatory, endothelial, hepatic, fibrotic, and metabolic perturbations rather than isolated organ failure endpoints^2,4^.

Mitochondrial dysfunction has also emerged as an important component of the shared CKM pathobiology. Increasing evidence indicates that impaired mitochondrial energetics, excessive oxidative stress, altered substrate utilization, and reduced metabolic flexibility contribute to progressive myocardial and renal dysfunction by promoting inflammation, endothelial injury, and profibrotic signaling. These mitochondrial alterations may represent a common downstream pathway linking metabolic disease to cardiovascular and kidney injury, thereby reinforcing the concept of CKM syndrome as a systems-level disorder rather than the coexistence of isolated organ diseases. Recent mechanistic studies suggest that modulation of mitochondrial function may contribute to the pleiotropic cardiorenal effects observed with contemporary CKM therapies, providing additional biological plausibility for their clinical benefits^2,4,35^ (Figure 1).

**Figure 1:**
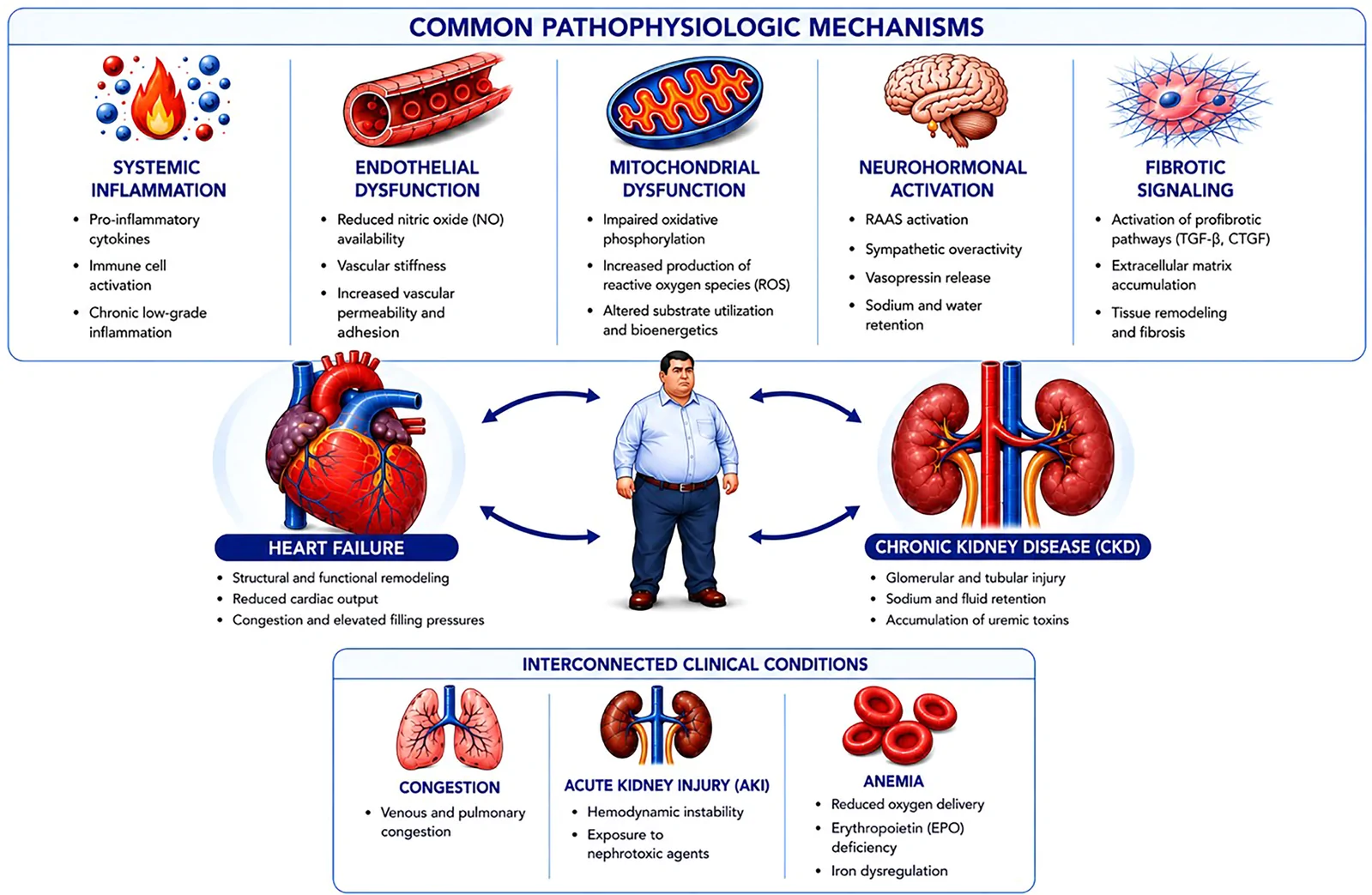
Shared pathophysiologic mechanisms and clinical manifestations of cardiovascular-kidney-metabolic syndrome.

### SGLT2 inhibitors in 2025: earlier use, broader contexts, and mechanistic correlates

SGLT2 inhibitors remained foundational for CKM risk modification, and 2025 evidence expanded the class’s evidence base regarding timing, clinical context, and mechanistic correlates.

### Initiation during hospitalization for heart failure

The DAPA ACT HF–TIMI 68 trial evaluated in-hospital initiation of dapagliflozin in patients hospitalized for heart failure but did not demonstrate a statistically significant reduction in its primary composite endpoint at 60 days. However, the accompanying prespecified meta-analysis, which integrated DAPA ACT HF–TIMI 68 with previous randomized trials of early in-hospital SGLT2 inhibitor initiation, demonstrated consistent reductions in cardiovascular death and worsening heart failure. These findings support the safety and potential clinical value of initiating SGLT2 inhibitors during hospitalization while recognizing that this conclusion is derived from the totality of the evidence rather than from the standalone DAPA ACT HF–TIMI 68 trial^36^.

### Severe HF and treatment effects across the EF spectrum

Analyses spanning DAPA-HF and DELIVER addressed severe HF populations and treatment effects across EF strata^37^. Additional work interrogated heterogeneity by achieved EF, reinforcing that clinical risk may remain substantial even when EF improves and that de-intensification based solely on “improved EF” may be discordant with ongoing risk biology^38^. Methodologic work examining approaches to minimize risk heterogeneity bias in HF trials further highlighted how analytic framing can influence interpretation of treatment effects and residual risk^9^. In aggregate, these findings reinforce an important operational point for CKM care: therapy should track biologic and clinical vulnerability, not only EF labels or perceived recovery^9,37,38^.

### Biomarkers, metabolic signatures, and biological plausibility

Mechanistic and biomarker analyses advanced hypotheses for SGLT2 inhibitor benefits. The EMPEROR program work evaluated erythropoiesis and iron mobilization pathways with empagliflozin in HF, supporting a plausible link between SGLT2 inhibition and improved hematologic substrate delivery that may influence symptoms and functional status^39^. Exploratory analyses in CKD populations investigated uric acid lowering as a metabolic phenotype potentially relevant to vascular and renal risk^40^, while acute HF data examined uric acid trajectories during recompensation^41^. *Post hoc* analyses further evaluated iron metabolism signals within SGLT2 inhibitor trials in CKD^42^. Biomarker-focused studies assessed empagliflozin effects in HF patients with type 2 diabetes^37^ and fibrosis-related biomarkers, including galectin-3^43^, in dapagliflozin-treated diabetes populations^44^. Mechanistic clinical work in diabetes explored cellular and podocyte-related markers supporting renal-protective plausibility^45^, and experimental data linked SGLT2 inhibition to renal branched-chain amino acid catabolism^46^.

An important evolution in this field has been the increasing availability of centralized biorepositories linked to large randomized trials, enabling more granular analyses that extend beyond cross-sectional associations between disease severity and biomarker levels to include dynamic biomarker responses to active treatment vs. placebo^28,29,31,39^. This shift allows biomarkers to be interpreted not only as markers of risk but also as indicators of biological engagement and therapeutic modulation.

In parallel, insights from protocol-driven kidney biopsies in well-characterized populations—such as CKD related to diabetes and hypertension, as well as acute kidney injury—have further enriched mechanistic understanding^46^. Initiatives such as the Precision Kidney Medicine Project (https://www.kpmp.org/) exemplify how integrated molecular, histopathologic, and clinical data can refine phenotype definitions and link biomarker signatures to structural injury and repair pathways.

Together, these mechanistic strands strengthen biologic plausibility for multi-organ benefit and help interpret “class effects” as convergent downstream modulation of inflammation, metabolism, and tissue remodeling^39,40,41,42,43,44,45,46,47^.

### Post-myocardial infarction cardiometabolic prevention

Subanalyses of DAPA-MI examined cardiometabolic outcomes after acute MI by baseline glycemic status, body mass index (BMI), and EF, aligning with a preventive CKM paradigm emphasizing intervention along high-risk trajectories rather than waiting for established HF or progressive CKD^48,49^. Mechanistic framing work linking early post-MI SGLT2 inhibition to inflammatory and senescence pathways further supports preventive positioning^27^. Collectively, the 2025 evidence base continues to shift SGLT2 inhibitors from “downstream HF/CKD therapy” toward earlier CKM trajectory modification in selected high-risk contexts^27,48,49^.

### Contextual evidence in acute systemic illness

Prespecified analyses of DARE-19 evaluated dapagliflozin in patients hospitalized with COVID-19 with and without type 2 diabetes, adding context-specific safety and outcome data for SGLT2 inhibition in acute illness settings^50^. These data reinforce the importance of defining not only who benefits but also when and under what acute physiologic constraints therapy can be safely initiated and maintained^50^.

### Consolidation of nonsteroidal MRA (nsMRA) evidence across CKM phenotypes

In 2025, nsMRA emerged as a central CKM therapy, particularly through the FINEARTS-HF program and its prespecified analyses addressing kidney outcomes, phenotypic heterogeneity, and safety-relevant electrolyte biology.

A major contribution was the evaluation of kidney outcomes in FINEARTS-HF, addressing a historically uncertain evidence domain—renal endpoints in HF populations with varying degrees of CKD and diabetes burden^10^. Prespecified analyses clarified how early eGFR declines with finerenone should be interpreted in clinical practice, framing the initial renal trajectory as part of the expected pharmacodynamic profile rather than necessarily a signal of harm^51^. This is clinically consequential: it supports continued therapy in appropriate patients when early eGFR changes occur in predictable patterns and are contextualized within longer-term risk-benefit tradeoffs^10,51^.

### Efficacy across EF and clinically relevant subgroups

Prespecified analyses evaluated finerenone across the EF spectrum^52^, in patients with recent worsening HF events^53^, and among individuals with improved EF^54^. Additional subgroup analyses addressed frailty^55^, obesity^56^, atrial fibrillation^16^, and sex-related differences in treatment effect^17^. Patient-centered outcomes were assessed via disease-specific health status instruments, including KCCQ (Kansas City Cardiomyopathy Questionnaire) analyses^18^, and long-term benefit estimation was explored using prespecified modeling approaches^19^. Risk-model contextualization using PREDICT-HFpEF further supports a phenotype-informed approach to nonsteroidal MRA deployment^20^. Beyond HF, mechanistic trial data examined arterial stiffness and cardiorenal biomarkers in type 2 diabetes with CKD^57^, reinforcing vascular and renal plausibility. Taken together, the FINEARTS-HF program and its analyses strengthen finerenone’s position as a phenotype-relevant CKM therapy whose value extends beyond a single trial headline into interpretable, patient-centered, and clinically stratified benefits^16,17,18,20,53,54,55,56,57,58,59,60^.

### Safety, potassium biology, and therapy enablement strategies

Electrolyte management remained clinically central. A FIDELITY analysis evaluated hypokalemia patterns and outcomes in patients with type 2 diabetes and CKD treated with finerenone^61^. In parallel, HF management studies examined strategies to enable optimized MRA therapy among hyperkalemia-prone patients; sodium zirconium cyclosilicate was studied as an approach to facilitate spironolactone optimization^62^. Dialysis populations were addressed by a large randomized trial of spironolactone in maintenance dialysis, informing the nuanced role of steroidal MRA in advanced kidney disease^63^. Additional analyses explored aldosterone-MRA interaction patterns in EPHESUS^64^, and pharmacokinetic work examined balcinrenone in severe renal impairment^11^, complementing the broader MRA landscape relevant to CKM populations. Collectively, these data emphasize that “MRA implementation” is increasingly a science of enablement—risk stratification, monitoring, and mitigation strategies—rather than a binary decision to treat or not treat^11,14,62,63,64^.

### Combination therapy: finerenone and SGLT2 inhibition

The CKM paradigm emphasizes combined pathway blockade to reduce residual risk. The CONFIDENCE trial baseline report characterized participants and an albuminuria-focused endpoint^65^, and a subsequent analysis evaluated simultaneous initiation of finerenone and empagliflozin across kidney-risk strata^66^. In HF, analyses evaluated finerenone effects with and without concomitant SGLT2 inhibitor use^19^. A dedicated strategy trial tested SGLT2 inhibition with and without aldosterone antagonism in HFpEF (SOGALDI-PEF), directly interrogating the combination strategy within a phenotype-rich syndrome class^67^. MIRO-CKD described trial design and baseline characteristics evaluating balcinrenone and dapagliflozin in CKD^68^, and additional CKD subgroup analyses addressed finerenone efficacy and safety across kidney function strata in Asian patients^69^. Together, 2025 marks a transition from conceptual enthusiasm about combination CKM therapy to systematic evaluation—across outcomes, mechanistic endpoints, and practical initiation strategies^3,4,19,65,66,67,68,69^.

### Incretin-based therapy and obesity-driven CKM phenotypes

Obesity and dysglycemia remain dominant drivers of CKM expression, particularly in HFpEF. The SUMMIT trial analysis examined how CKD modifies the effects of tirzepatide in patients with HFpEF and obesity—an increasingly common phenotype in contemporary practice where adiposity, renal vulnerability, and congestion coexist^12^. In parallel, cardiovascular outcomes analyses of glucagon-like peptide-1 (GLP-1) receptor agonists continued to explore heterogeneity by EF, leveraging EXSCEL-derived analyses^14^. Mechanistic work investigated myocardial glucose uptake and markers of diastolic dysfunction during GLP-1/glucagon co-infusion in adults with type 2 diabetes^15^. Additional clinical studies evaluated the effects of dulaglutide alone vs. those of dulaglutide plus probiotics on cardiometabolic risk factors^13^, and prespecified analyses of the SOUL trial examined oral semaglutide cardiovascular outcomes stratified by background SGLT2 inhibitor use^8^.

More recently, tirzepatide demonstrated cardiovascular safety by proving noninferior to dulaglutide for major adverse cardiovascular events (MACE) while maintaining its robust effects on glycemic control and body weight. Although a numerical reduction in events was observed, superiority was not achieved, positioning tirzepatide as a metabolically potent therapy with confirmed cardiovascular safety and reinforcing its role within the evolving cardiovascular-kidney-metabolic treatment landscape^70^.

Emerging mechanistic insights also come from the REMODEL trial, which highlights how incretin receptor modulation impacts myocardial and adipose tissue signaling pathways relevant to remodeling, metabolism, and systemic inflammation in obesity-driven CKM phenotypes^71^.

Collectively, 2025 supports an integrated CKM narrative in which incretin-based strategies increasingly sit alongside SGLT2 inhibition and MRA therapy—particularly in obesity-linked HF phenotypes where weight trajectory, congestion biology, and functional limitation intersect^12,52^.

### Amylin analogues and the cardiovascular-kidney-metabolic *continuum*

Amylin analogs are synthetic peptides that mimic the actions of amylin, a hormone co-secreted with insulin by pancreatic β-cells in response to food intake. Amylin acts primarily within the central nervous system, particularly the area postrema, promoting satiety, reducing appetite, delaying gastric emptying, and suppressing postprandial glucagon secretion, thereby contributing to glycemic control and body-weight regulation. Cagrilintide is a long-acting amylin analog under investigation for obesity management, with potential benefits across cardiovascular, renal, and metabolic pathways. Phase 2 studies demonstrated that cagrilintide induces clinically meaningful weight loss (up to 10.8% at 26 weeks) and improves metabolic parameters, with a safety profile comparable to that of liraglutide^72^. These findings were extended in the phase 3a REDEFINE 1 and 2 trials, in which the fixed-dose combination of semaglutide/cagrilintide (CagriSema) produced substantial weight loss and clinically relevant reductions in systolic and diastolic blood pressure and lipid profile, supporting its role in integrated cardiovascular-kidney-metabolic risk reduction^73,74^.

### Lipids, atherosclerosis, and vascular risk in CKM syndrome

Atherosclerotic disease remains a principal contributor to CKM outcomes. The PURSUIT randomized trial evaluated an oral PCSK9 inhibitor for hypercholesterolemia, addressing adherence and access barriers inherent to injectable therapies and aligning with implementation-driven vascular prevention priorities^75^. Additional analyses from ODYSSEY OUTCOMES focused on high-risk dyslipidemia phenotypes, including probable familial hypercholesterolemia or type III hyperlipoproteinemia^76^. Lipoprotein(a) was evaluated as a vascular risk marker in chronic limb-threatening ischemia after revascularization, with attention to major adverse limb events and mortality^77^. Beyond lipids, a phase 2 trial targeting LOX-1 inhibition in patients with residual inflammation after MI signaled continued interest in anti-inflammatory vascular strategies potentially relevant to CKM populations with persistent inflammatory activation^78^.

More recently, the ZEUS trial extended this paradigm by directly targeting interleukin-6 signaling with ziltivekimab in patients with chronic kidney disease and established atherosclerotic cardiovascular disease, demonstrating significant reductions in inflammatory biomarkers and providing proof of concept for cytokine-directed vascular risk modification in CKM populations characterized by heightened inflammatory burden (NCT05021835).

These vascular data reinforce a core CKM principle: integrated risk modification must encompass both hemodynamic/renal pathways and atherosclerotic-inflammatory biology, particularly in patients with overlapping cardiorenal vulnerability and residual vascular risk^75,76,77,78^.

### Iron deficiency, anemia, and the kidney-heart axis

Iron deficiency and anemia remain prominent and potentially modifiable contributors to symptoms, functional status, and hospitalization risk across HF and CKD^79^. The FAIR-HF2 DZHK05 randomized clinical trial addressed uncertainty regarding intravenous ferric carboxymaltose in HF with iron deficiency^80^, and baseline characteristics analyses contextualized the enrolled population relative to prior iron trials^81^. IRONMAN provided further insights into infection risk with iron repletion^82^ and age-stratified effects of intravenous ferric derisomaltose^83^. In renal anemia, a pilot randomized study evaluated daprodustat in HF with renal anemia and examined relationships between hemoglobin response and iron metabolism markers such as hepcidin^84^. Together, these studies highlight that the CKM agenda increasingly includes hematologic and metabolic co-drivers of congestion, exercise intolerance, and clinical instability—domains that meaningfully influence outcomes and quality of life yet often remain undertreated in conventional cardiorenal care^39,80,81,82,83,84^.

### Congestion, hemodynamics, and acute kidney injury across CKM settings

The tension between achieving effective decongestion and preserving kidney function remains central in acute HF and perioperative settings. The DECONGEST study rationale described a strategy of serial spot urine sodium-guided diuretic therapy in acute HF, aiming to improve decongestion efficiency and safety^25^. Relaxin-pathway therapies continued to be studied in preserved-EF worsening HF: a phase 2 trial of volenrelaxin examined left atrial function, congestion, and kidney function^23^, while RELAX-AHF-2 biomarker substudy results explored end-organ protective signals^85^. Early-phase work evaluated renal perfusion effects of a relaxin receptor agonist in HF with reduced EF (HFrEF)^86^, and a mechanistic combination study examined AZD5462 on top of dapagliflozin in HF with moderate renal impairment^87^.

Acute kidney injury (AKI) prevention and recovery were investigated in cardiac surgery settings with ANG-3777^88^ and in trials evaluating anesthetic strategies and renal function endpoints^89,90^. Collectively, these studies reflect ongoing maturation of a CKM approach to acute illness: congestion, perfusion, inflammation, and renal injury are increasingly examined as interconnected and modifiable processes, rather than inevitable collateral damage of decongestion or complex procedures^24,25,77^.

Despite the maturation of the CKM framework, patients receiving maintenance dialysis remain systematically underrepresented across pivotal cardiovascular, kidney, and metabolic trials^1,2,63^. This persistent exclusion reflects a convergence of methodological, regulatory, and biological concerns, including heightened competing risks, altered pharmacokinetics, safety fears related to electrolyte instability and hypotension, and uncertainty regarding appropriate endpoints in a population in which mortality, hospitalization, and quality of life frequently diverge from earlier CKM trajectories^63^. As a result, dialysis populations have often been treated as a boundary condition rather than as a *continuum* within CKM biology, reinforcing a clinical evidence gap that is disproportionate to their absolute cardiovascular and metabolic risk burden^1,2^.

### Risk stratification: from biomarkers to frailty, adiposity, and machine learning

Risk prediction and patient selection continued to shift from single-axis severity grading toward integrated phenotype- and trajectory-based frameworks. A validated multivariable machine-learning model was developed to predict cardiorenal risk in diabetic kidney disease using clinical variables and biomarkers, illustrating the growing role of data-driven tools to guide intensification and combination therapy^91^. In HF populations, nutritional scores were evaluated for prognostic value^92^, and temporal changes in biomarkers, function, and quality of life were mapped in advance of adverse outcomes in HF with mildly reduced or preserved EF (HFmrEF/HFpEF)^93^. Wearable accelerometer-derived physical activity measures were studied as potential digital biomarkers in HF trial populations^52^. Importantly, adiposity-related anthropometrics and obesity metrics were evaluated in pooled HFpEF/HFmrEF analyses with kidney outcomes and clinical endpoints, supporting more granular characterization of adiposity beyond BMI alone^94,95^. Complementary work in diabetes evaluated inflammatory markers and cardiovascular autonomic neuropathy measures, emphasizing that inflammatory and neuroautonomic domains may further refine phenotyping in dysglycemic CKM populations^96^. Taken together, these studies support an expanded CKM phenotype definition incorporating functional status, frailty, adiposity distribution, biomarker trajectories, and digital phenotypes—dimensions likely to improve alignment between biologic risk and therapeutic strategy^4,91,94^.

### Lifestyle and dietary interventions: emerging, but still early

Lifestyle interventions received targeted attention, often incorporating digital augmentation and metabolomic profiling. A pilot randomized trial compared a digitally enhanced ketogenic diet vs. a low-fat diet for cardiovascular-kidney-metabolic health in a predominantly Hispanic population with overweight/obesity, integrating metabolomics and digital health elements^96^. Other work evaluated dietary or supplement-based strategies such as glycocalyx mimetics in type 2 diabetes using urinary peptidomic classifiers^97^, and exploratory physiology studies examined strategies to mitigate AKI-associated markers during heat exposure^35^. Recent evidence has also drawn attention to the detrimental role of ultra-processed foods (UPFs) in CKM syndrome. Beyond their high content of refined carbohydrates, unhealthy fats, sodium, and food additives, UPFs have been consistently associated with systemic inflammation, oxidative stress, insulin resistance, dyslipidemia, immune dysregulation, and alterations in the gut microbiota, mechanisms that collectively promote cardiovascular, kidney, and metabolic disease progression^98^. These findings reinforce that dietary recommendations in CKM syndrome should emphasize not only caloric restriction and weight reduction but also improvements in overall dietary quality through prioritizing minimally processed dietary patterns while limiting ultra-processed food consumption. While scientifically informative, these studies remain largely hypothesis-generating relative to pharmacotherapy outcome trials, underscoring the need for larger, adequately powered, outcome-driven evaluations of lifestyle-based CKM care pathways^97,99,100^.

The major advances reviewed throughout 2025 converge toward an integrated CKM framework in which shared biology, disease-modifying therapies, and phenotype-guided implementation collectively redefine cardiovascular-renal-metabolic care (Figure 2).

**Figure 2:**
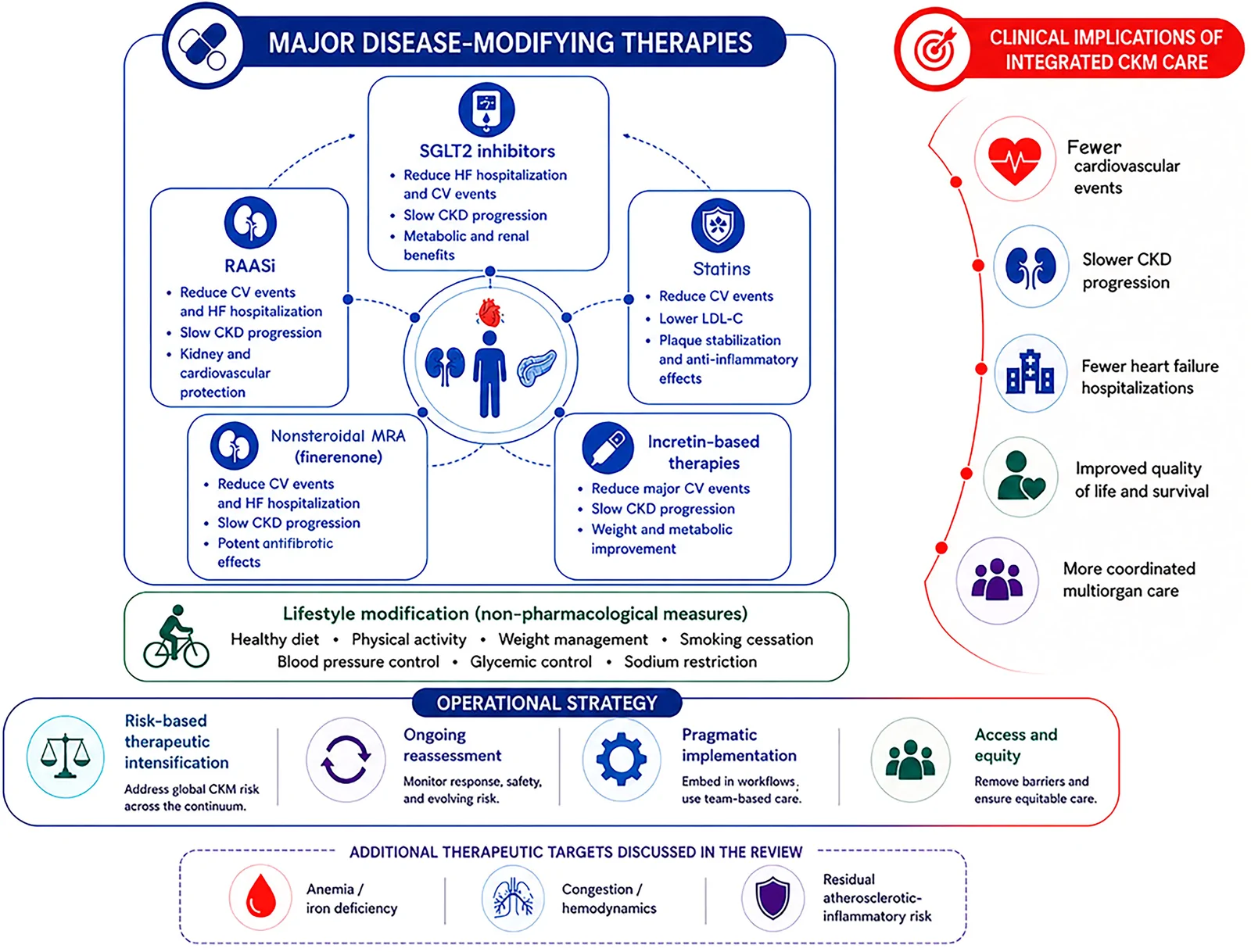
Contemporary management of cardiovascular-kidney-metabolic syndrome: disease-modifying therapies and clinical implications.

### Implications for clinical practice and research priorities

In 2025, the CKM syndrome paradigm moved further from concept toward operational strategy. First, evidence supported earlier and more consistent deployment of foundational therapies, including SGLT2 inhibitors initiated during HF hospitalization, with data consistent with cardiometabolic prevention early after MI^36,48,87^. Second, the evidence base increasingly supported rational combination therapy to reduce residual risk, particularly through expansion of finerenone data in HF and kidney outcomes and through trials and analyses evaluating or contextualizing concurrent use with SGLT2 inhibitors^19,65,66,67^. Third, care increasingly gravitated toward phenotype-driven interpretation beyond single-organ labels, as frailty, adiposity distributions, biomarker trajectories, machine-learning risk tools, and patient-reported outcomes became central to treatment targeting and effect modification^18,55,91,93,94^. Finally, important uncertainty persists in advanced CKD and acute-care settings, where dialysis populations, perioperative AKI, and decongestion strategies in acute HF remain active areas with heterogeneous practice patterns and incomplete out­comes evidence^63,88,101^.

Looking forward, three priorities emerge: (1) definitive outcomes trials of rational combination regimens in carefully phenotyped CKM populations are needed to move beyond extrapolation and toward truly personalized pathway layering^3,4,91^; (2) pragmatic implementation studies must address adoption gaps—initiation timing, sequencing, persistence, monitoring, and equity—because the magnitude of benefit in trials will not translate if therapy is delayed, discontinued, or inconsistently deployed across systems^1,3^; (3) the safe and equitable integration of digital biomarkers, omics, and machine-learning decision support into real-world workflows should be prioritized, with careful attention to bias, generalizability, and clinical accountability^4,91^. The overarching implication of 2025 is that CKM care is increasingly defined by earlier intervention, combination strategies, and phenotype-guided application, balanced against implementation constraints and the persistent evidence gaps at the extremes of kidney disease and acute illness^1,3,4,102^.

### Methodological considerations and interpretive caution

While the 2025 CKM literature substantially advanced therapeutic integration and mechanistic understanding, it is important to acknowledge that not all randomized evidence yielded unequivocally positive results. Several contemporary trials and prespecified analyses demonstrated neutral effects on hard outcomes such as all-cause or cardiovascular mortality, despite favorable signals in secondary endpoints, biomarkers, or patient-reported outcomes^9,63,80,88^. These findings underscore the persistent challenge of residual risk in advanced CKM states and caution against overinterpreting surrogate or intermediate endpoints as definitive evidence of disease modification—particularly in populations characterized by high competing risk, short follow-up, or constrained sample size.

A broader methodological consideration relates to the potential influence of publication bias and analytic fragmentation within the expanding CKM evidence base. Positive subgroup analyses, post hoc biomarker studies, and phenotype-enriched signals may preferentially reach publication, whereas neutral or negative findings—especially those related to therapeutic sequencing, combination strategies, or implementation failure—remain underrepresented^63,80^. Moreover, the dissemination of prespecified and exploratory analyses across overlapping trial populations risks fragmenting interpretation and inflating the perceived consistency of benefit. Recognizing these limitations is essential to avoid premature extrapolation and to ensure that the CKM framework evolves with appropriate scientific humility, balancing enthusiasm for pathway-based integration with rigorous appraisal of uncertainty, heterogeneity, and external validity^9,63,80,88^.

## Data Availability

The datasets generated and/or analyzed during the current study are available from the corresponding author upon reasonable request.
