# Supplementary material for: The cardiovascular-kidney-metabolic syndrome in 2025: a year in review
Source: J Bras Nefrol. 2026 Nov 9;48(4):e20260052. doi: 10.1590/2175-8239-JBN-2026-0052en (PMC13747300; doi:10.1590/2175-8239-JBN-2026-0052en)
Supplement: Translation

# Translation: A síndrome cardiovascular-renal-metabólica em 2025: uma revisão anual

**DOI:** 10.1590/2175-8239-JBN-2026-0052pt

**Authors:** Rogerio Passos, Ana Flávia Moura, Thyago Proença de Moraes, Felipe Costa Neves, Luis Claudio Santos Pinto, Fabiano Bichuette Custodio, José A. Moura-Neto, Roberto Pecoits-Filho

**Affiliations:** 1 Sociedade Brasileira de Nefrologia, São Paulo, SP, Brasil.; 2 DaVita Brasil, São Paulo, SP, Brasil.; 3 Hospital Israelita Albert Einstein, São Paulo, SP, Brasil.; 4 Escola Bahiana de Medicina e Saúde Pública, Salvador, BA, Brasil.; 5 Pontifícia Universidade Católica do Paraná, Escola de Medicina, Curitiba, PR, Brasil.; 6 Hospital Ana Nery, Salvador, BA, Brasil.; 7 Universidade Federal do Pará, Belém, PA, Brasil.; 8 Universidade Federal do Triângulo Mineiro, Uberaba, MG, Brasil.; 9 University of Michigan, Division of Nephrology, Ann Arbor, Estados Unidos.; 10 Arbor Research Collaborative for Health, ClinEpi Program, Ann Arbor, United States.

**Keywords:** Síndrome Metabólica, Insuficiência Renal Crônica, Implementação de Plano de Saúde, Fatores de Risco de Doenças Cardíacas, Doenças Metabólicas

## Abstract

**Introdução:**

A síndrome cardiovascular-renal-metabólica (SCRM) surgiu como uma estrutura clínica integrada que reflete uma fisiopatologia compartilhada e trajetórias de risco sobrepostas entre as doenças cardiovascular, renal e metabólica. Em 2025, esse paradigma avançou de um constructo conceitual para uma estratégia operacional, fundamentada em ensaios clínicos randomizados (ECR), estudos mecanísticos e análises voltadas à implementação.

**Principais achados:**

Esta revisão sintetiza os principais ECR com análises pré-especificadas, análises secundárias, estudos mecanísticos e relatórios de desenho de estudo publicados durante 2025 que moldam os cuidados contemporâneos em SCRM. As evidências consolidaram os iSGLT2 e os antagonistas não esteroidais do receptor mineralocorticoide como terapias fundamentais nos fenótipos de insuficiência cardíaca (IC) e doença renal crônica (DRC), com dados crescentes sobre início mais precoce, estratégias de combinação e aplicação guiada por fenótipo. As terapias baseadas em incretinas abordaram os fenótipos de SCRM impulsionados pela obesidade, particularmente a IC com fração de ejeção preservada, enquanto ensaios mecanísticos e análises de biomarcadores reforçaram a plausibilidade biológica nas vias inflamatória, endotelial, metabólica e fibrótica. Os avanços na prevenção vascular destacaram a relevância do risco inflamatório e aterosclerótico residual, além da redução lipídica, e estudos direcionados à anemia, à congestão e à lesão renal aguda ampliaram o escopo operacional do manejo da SCRM. Desenvolvimentos paralelos na estratificação de risco incorporaram fragilidade, adiposidade, trajetórias de biomarcadores e ferramentas de aprendizado de máquina, apoiando uma transição de limiares de gravidade de órgão único para um cuidado integrado, baseado em trajetórias.

**Conclusões:**

A base de evidências de 2025 reforça a SCRM como um estado de doença em nível sistêmico, abordado por meio de intervenção mais precoce, estratificação racional de vias terapêuticas e modelos de cuidado integrado. As prioridades futuras incluem ensaios definitivos de desfechos com estratégias combinadas, estudos pragmáticos de implementação e melhor inclusão de populações com DRC avançada e em diálise, para assegurar a tradução equitativa dos avanços em SCRM para a prática clínica.

## INTRODUÇÃO

Ao longo de 2025, a síndrome cardiovascular-renal-metabólica (SCRM) evoluiu de um rótulo descritivo de comorbidades agrupadas para uma estrutura clínica mais acionável, com implicações diretas para prevenção, rastreamento, definição de fenótipos, estratificação de risco e sequenciamento terapêutico^1^. É importante ressaltar que essa evolução reflete uma mudança em direção a um constructo holístico e multiorgânico, em vez de silos específicos de doença, posicionando a SCRM como um *continuum* que abrange os domínios cardiovascular, renal e metabólico.

Três temas convergentes foram repetidamente reforçados. Primeiro, a insuficiência cardíaca (IC), a doença cardiovascular aterosclerótica, a doença renal crônica (DRC) e a disglicemia/obesidade compartilham uma fisiopatologia sobreposta — inflamação sistêmica, disfunção endotelial, sinalização fibrótica mal-adaptativa e utilização alterada de substratos — que plausivelmente impulsiona trajetórias paralelas de lesão orgânica e instabilidade clínica^2^. Segundo, o início mais precoce e a estratificação racional de terapias modificadoras da doença, associados a modificações abrangentes no estilo de vida, são cada vez mais necessários para mitigar o risco residual que persiste quando o cuidado permanece ancorado à gravidade de órgão único, à doença em estágio avançado ou a paradigmas de tratamento reativo^3^. Terceiro, a avaliação de risco em SCRM está avançando além dos limiares de taxa de filtração glomerular estimada (TFGe) ou de fração de ejeção (FE) em direção a uma fenotipagem multidimensional, integrando biomarcadores, fragilidade, métricas relacionadas à adiposidade e abordagens de aprendizado de máquina, para melhor alinhar a intensidade terapêutica ao risco biológico^4^. Em conjunto, esses temas ressaltam a crescente necessidade de modelos de cuidado integrados e longitudinais que coordenem estratégias de prevenção e tratamento entre especialidades e estágios da doença, em vez de um manejo fragmentado e específico por órgão.

A crescente relevância clínica da SCRM vai além de sua complexidade fisiopatológica e reflete-se cada vez mais em sua carga em nível populacional. A SCRM foi responsável por mais de 3,3 milhões de óbitos nos Estados Unidos entre 2010 e 2019, com mortalidade aumentando de forma constante ao longo da década e disparidades marcantes conforme idade, raça e região geográfica^5^. Da mesma forma, um estudo populacional de âmbito nacional realizado na China demonstrou a alta prevalência da SCRM e sua estreita associação com desfechos clínicos adversos, destacando que esse constructo emergente representa um desafio de saúde global que se estende bem além das populações ocidentais^6^. Complementando essas observações, dados prospectivos do UK Biobank demonstraram um aumento gradual da mortalidade por todas as causas, cardiovascular, por câncer e por outras causas ao longo dos estágios avançados da SCRM, mostrando também que a adesão a um estilo de vida saudável atenuou substancialmente esses riscos, independentemente do estágio da doença^7^. Em conjunto, esses achados reforçam o crescente impacto da SCRM em nível populacional e ressaltam a necessidade de estratégias integradas de prevenção, intervenção precoce e avaliação contínua dos avanços terapêuticos emergentes.

Os ensaios e análises pré-especificadas do ano consolidaram ainda mais as evidências sobre os inibidores do cotransportador sódio-glicose tipo 2 (iSGLT2)^8,9^, os antagonistas não esteroidais do receptor mineralocorticoide (ARMne) — como finerenona^10,11^ — e as estratégias baseadas em incretinas nos fenótipos de IC relacionados à obesidade^12,13,14,15^. Paralelamente, programas emergentes direcionados à anemia^16,17,18,19,20^, ao manejo da congestão e da hemodinâmica^21,22,23,24,25^ e à redução da albuminúria como desfecho terapêutico^26^ ampliaram o escopo operacional do cuidado em SCRM. Nesta revisão, adotamos uma perspectiva ampla de SCRM para sintetizar os principais ensaios clínicos randomizados (ECR) publicados em 2025, enfatizando como as intervenções integradas embasam a prevenção e o manejo contemporâneos no espectro cardiovascular-renal-metabólico, em vez de priorizar uma visão de órgão único. A Tabela 1 resume os principais estudos clínicos publicados durante 2025, incluindo ECR marcantes, juntamente com análises pré-especificadas influentes, estudos mecanísticos e relatórios de desenho de estudo que moldaram a evolução da SCRM.

**Tabela 1:** Principais ensaios clínicos randomizados e análises-chave publicados em 2025 que moldaram o conceito da síndrome cardiovascular-renal-metabólica (SCRM).

Estudo	Tipo de publicação (2025)	*N*	População	Intervenção	Desfecho primário	Principais achados	Relevância clínica
SOUL^8^	Análise pré-especificada	9.650	Adultos ≥50 anos com DM2 e DAC aterosclerótica estabelecida e/ou DRC	Semaglutida oral vs. placebo	MACE	Redução consistente de MACE independentemente do uso concomitante de iSGLT2	Confirma o benefício cardiovascular dos agonistas do receptor de GLP-1 orais
SUMMIT^12^	Ensaio clínico randomizado primário	731	ICFEp e obesidade	Tirzepatida	Morte CV ou piora da IC	Redução do desfecho CV composto e melhora do estado de saúde	Estabelece a terapia direcionada à obesidade como estratégia para ICFEp
FLOW^103^	Análise pré-especificada	3.533	DM2 e DRC	Semaglutida	Desfechos renais e CV	Proteção renal e CV consistente em subgrupos clinicamente relevantes	Reforça os agonistas do receptor de GLP-1 como terapia nefroprotetora
DECODE-CKD^104^	Ensaio clínico randomizado mecanístico	222	DRC	Dapagliflozina	Mudança no índice de massa ventricular esquerda	Redução significativa do índice de massa ventricular esquerda	Fornece evidências mecanísticas de remodelamento reverso cardíaco
MIRO-CKD^17^	Relato do desenho do estudo e das características basais	324	DRC com albuminúria	Balcinrenona + dapagliflozina	Mudança na RACU	Racional do estudo e características basais relatados	Avalia a inibição combinada do receptor mineralocorticoide e de SGLT2
CONFIDENCE^64^	Relato do desenho do estudo e das características basais	807	DM2 e DRC	Finerenona + empagliflozina	Mudança na RACU	Racional do estudo e características basais relatados	Apoia a inibição combinada das vias da SCRM
DAPA ACT HF–TIMI 68^30^	Ensaio clínico randomizado primário	2.401	Hospitalização por IC aguda	Dapagliflozina	Morte CV ou piora da IC	Desfecho primário neutro; metanálise concomitante sustentou o início precoce do tratamento durante a hospitalização	Reforça o início precoce da terapia com iSGLT2 com base no conjunto das evidências
FINEARTS-HF^47^	Análises pré-especificadas	6.001	ICFElr/ICFEp	Finerenona	Múltiplos desfechos CV e renais pré-especificados	Ampliação das evidências em fenótipos clinicamente relevantes da SCRM	Amplia o papel dos ARMne em todo o espectro da SCRM
FAIR-HF^251^	ECR primário	1.105	ICFEr com deficiência de ferro	Carboximaltose férrica intravenosa	Mortalidade CV ou hospitalização por IC	Desfecho primário neutro, com melhora da capacidade funcional	Apoia a correção individualizada da deficiência de ferro
IRONMAN^55^	Análises secundárias	1.137	IC com deficiência de ferro	Derisomaltose férrica intravenosa	Desfechos de eficácia e segurança estratificados por idade	Eficácia e segurança consistentes nos subgrupos clinicamente relevantes	Apoia a terapia intravenosa individualizada com ferro
SURPASS-CVOT^69^	ECR primário de desfechos CV	13.299	DM2 com DCVA estabelecida	Tirzepatida	MACE	Demonstrou segurança CV em comparação com a dulaglutida	Reforça o perfil de segurança CV da tirzepatida
REDEFINE ^172^	Ensaio clínico randomizado primário	3.417	Obesidade	CagriSema	Variação relativa do peso corporal (coprincipal: perda de peso ≥5%)	Redução de peso superior em comparação ao placebo	Fortalece a terapia combinada baseada em incretinas para o tratamento da obesidade
REDEFINE ^273^	Ensaio clínico randomizado primário	1.206	DM2 e obesidade	CagriSema	Variação relativa do peso corporal	Redução de peso superior	Amplia as evidências para o manejo da obesidade no DM2
ACHIEVE-^162^	Ensaio clínico randomizado primário	559	DM2	Orforgliprona oral	Redução da HbA1c	Controle glicêmico e redução de peso superiores	Amplia as opções terapêuticas orais baseadas em incretinas

Abreviaturas – ECR: ensaio clínico randomizado; DCVA: doença cardiovascular aterosclerótica; DAC: doença arterial coronariana; DRC: doença renal crônica; SCRM: síndrome cardiovascular-renal-metabólica; CV: cardiovascular; IC: insuficiência cardíaca; ICFElr: insuficiência cardíaca com fração de ejeção levemente reduzida; ICFEp: insuficiência cardíaca com fração de ejeção preservada; ICFEr: insuficiência cardíaca com fração de ejeção reduzida; MACE: eventos cardiovasculares adversos maiores; iSGLT2: inibidores do cotransportador sódio-glicose tipo 2; ARMne: antagonistas não esteroidais do receptor mineralocorticoide; DM2: diabetes mellitus tipo 2; RACU: relação albumina-creatinina urinária; HbA1c: hemoglobina glicada; GLP-1: peptídeo semelhante ao glucagon tipo 1.

Nota – A coluna “Tipo de publicação (2025)” identifica a natureza da publicação incluída nesta revisão (ensaio clínico randomizado primário, análise pré-especificada, análise secundária, estudo randomizado mecanístico ou relato do desenho/características basais). Para estudos cujos resultados principais foram publicados antes de 2025, foram incluídas apenas as análises ou relatórios publicados em 2025 que forneceram novas informações clinicamente relevantes. A relevância clínica resume a principal contribuição de cada publicação para a evolução do conceito da SCRM.

Em conjunto, a base de evidências de 2025 fortalece uma narrativa unificadora: a SCRM é cada vez mais manejada como uma biologia compartilhada, abordada por meio da modificação estratificada de vias, enquanto a fenotipagem e a implementação — como as terapias são selecionadas, iniciadas e mantidas — tornam-se tão centrais quanto o próprio desenvolvimento de fármacos^1,2,3,4^.

## MÉTODOS

Esta revisão foi conduzida como uma revisão narrativa estruturada da literatura sobre SCRM publicada entre 1º de janeiro e 31 de dezembro de 2025. Estudos relevantes foram identificados por meio de buscas no PubMed/MEDLINE e na Embase, complementadas por buscas manuais em importantes periódicos de cardiologia, nefrologia, diabetes (DM) e medicina interna. Artigos publicados online antes da impressão durante 2025 foram considerados elegíveis quando representavam a primeira divulgação pública dos achados do estudo.

Priorizaram-se ECR que avaliassem desfechos clinicamente relevantes. Análises pré-especificadas, análises secundárias, estudos randomizados mecanísticos e relatórios de desenho de estudo ou de características basais publicados no mesmo período também foram incluídos quando forneciam informações importantes sobre a evolução da estrutura da SCRM ou tinham implicações substanciais para a prática clínica contemporânea. Revisões narrativas, opiniões de especialistas, relatos de caso e estudos sem relevância direta para a SCRM não foram sistematicamente incluídos.

## RESULTADOS E DISCUSSÃO

### Convergência mecanística: inflamação, disfunção endotelial, disfunção mitocondrial, fibrose e metabolismo

Uma direção definidora na pesquisa de 2025 foi o refinamento de hipóteses mecanísticas que explicam por que intervenções desenvolvidas para o controle glicêmico ou a modulação neuro-hormonal produzem efeitos clinicamente relevantes na IC e na DRC, além de seus domínios canônicos. Trabalhos experimentais e translacionais propuseram que a inibição precoce de SGLT2 após infarto do miocárdio (IM) em pessoas com DM se relaciona com a ativação da imunidade inata — particularmente o inflamassoma NLRP3 — e com vias do fenótipo secretor associado à senescência, oferecendo uma estrutura anti-inflamatória e antissenescente coerente dentro da fisiopatologia da SCRM^27^. Paralelamente, análises exploratórias de grandes populações de ECR de IC sugeriram que mediadores inflamatórios, incluindo a interleucina-6, acompanham a progressão e os desfechos, de forma consistente com a inflamação funcionando tanto como marcador de risco quanto como mediador plausível de lesão orgânica subsequente^28,29^.

A disfunção endotelial continuou a emergir como um substrato unificador que liga a comorbidade cardiometabólica à IC em todo o espectro de FE. Análises multicoortes associaram alterações endoteliais à função cardíaca e aos desfechos clínicos, desde a saúde até a IC, alinhando-se à hipótese de que a inflamação impulsionada por comorbidades prejudica a função microvascular, amplifica a fisiopatologia da congestão e aumenta a vulnerabilidade renal — particularmente nos fenótipos de IC com FE preservada (ICFEp)^30^.

Análises proteômicas complementares em populações com DM e IC evidenciaram heterogeneidade biológica entre as categorias de FE, reforçando a visão de que a fisiopatologia relevante para a resposta terapêutica e o risco residual não se mapeia de forma clara apenas sobre a FE^31^.

Uma dimensão adicional desse paradigma multissistêmico em evolução é o reconhecimento crescente da doença hepática esteatótica associada à disfunção metabólica (MASLD) e da esteato-hepatite associada à disfunção metabólica (MASH) como importantes amplificadoras da SCRM. Em vez de representar um distúrbio hepático isolado, a MASLD/MASH é cada vez mais compreendida como parte de uma rede complexa de disfunção metabólica caracterizada por resistência à insulina, inflamação crônica de baixo grau, estresse oxidativo e fibrose progressiva, todos contribuindo para lesão cardiovascular e renal^32^. A gravidade da fibrose hepática tem sido associada de forma independente a desfechos cardiovasculares e renais adversos, sugerindo que o envolvimento hepático pode identificar ainda mais pacientes com risco cardiometabólico particularmente elevado. É importante destacar que ECR recentes mostraram que terapias já estabelecidas dentro da estrutura da SCRM também proporcionam benefícios hepáticos relevantes. A dapagliflozina melhorou significativamente características histológicas da MASH, incluindo resolução da esteato-hepatite e melhora da fibrose^33^, enquanto a semaglutida demonstrou melhoras substanciais tanto na resolução da esteato-hepatite quanto na fibrose hepática em pacientes com MASH confirmada por biópsia^34^. Esses achados reforçam o conceito de que direcionar vias metabólicas compartilhadas pode melhorar simultaneamente os desfechos hepáticos, cardiovasculares e renais, apoiando ainda mais uma abordagem integrada ao manejo da SCRM.

Em conjunto, os dados de 2025 ancoraram ainda mais a SCRM como um estado de doença em nível sistêmico, no qual os desfechos cardiorrenais refletem perturbações inflamatórias, endoteliais, hepáticas, fibróticas e metabólicas integradas, e não desfechos isolados de falência orgânica^2,4^.

A disfunção mitocondrial também emergiu como um importante componente da fisiopatologia compartilhada da SCRM. Evidências crescentes indicam que o comprometimento da bioenergética mitocondrial, o excesso de estresse oxidativo, a alteração da utilização de substratos energéticos e a redução da flexibilidade metabólica contribuem para a disfunção progressiva do miocárdio e dos rins, ao promover inflamação, lesão endotelial e sinalização pró-fibrótica. Essas alterações mitocondriais podem representar uma via final comum que conecta a doença metabólica às lesões cardiovascular e renal, reforçando o conceito da SCRM como um distúrbio sistêmico, e não como a simples coexistência de doenças isoladas de diferentes órgãos. Estudos mecanísticos recentes sugerem que a modulação da função mitocondrial pode contribuir para os efeitos cardiorrenais pleiotrópicos observados com as terapias contemporâneas para a SCRM, fornecendo plausibilidade biológica adicional para os benefícios clínicos dessas intervenções^2,4,35^ (Figura 1).

**Figura 1:** Mecanismos fisiopatológicos compartilhados e manifestações clínicas da síndrome cardiovascular-renal-metabólica (SCRM).

### Inibidores de SGLT2 em 2025: uso mais precoce, contextos mais amplos e correlatos mecanísticos

Os iSGLT2 permaneceram fundamentais para a modificação do risco em SCRM, e os dados de 2025 ampliaram a base de evidências da classe quanto ao momento de início, ao contexto clínico e aos correlatos mecanísticos.

### Início durante a hospitalização por insuficiência cardíaca

O ensaio DAPA ACT HF-TIMI 68 avaliou o início hospitalar da dapagliflozina em pacientes hospitalizados por IC, mas não demonstrou redução estatisticamente significativa em seu desfecho composto primário aos 60 dias. Entretanto, a metanálise pré-especificada que o acompanhou, integrando o DAPA ACT HF-TIMI 68 a ECR prévios de início hospitalar precoce de iSGLT2, demonstrou reduções consistentes na mortalidade cardiovascular e na piora da IC. Esses achados apoiam a segurança e o potencial valor clínico do início de iSGLT2 durante a hospitalização, reconhecendo-se que essa conclusão deriva da totalidade das evidências, e não apenas do ensaio DAPA ACT HF-TIMI 68 isoladamente^36^.

### IC grave e efeitos do tratamento ao longo do espectro da FE

Análises envolvendo os estudos DAPA-HF e DELIVER abordaram populações com IC grave e efeitos do tratamento entre os estratos de FE^37^. Trabalhos adicionais investigaram a heterogeneidade conforme a FE alcançada, reforçando que o risco clínico pode permanecer substancial mesmo quando a FE melhora e que a desintensificação baseada apenas na “FE melhorada” pode ser discordante da biologia de risco em curso^38^. Trabalhos metodológicos que examinaram abordagens para minimizar o viés de heterogeneidade de risco em ensaios de IC destacaram ainda mais como o enquadramento analítico pode influenciar a interpretação dos efeitos do tratamento e do risco residual^9^. Em conjunto, esses achados reforçam um ponto operacional importante para o cuidado em SCRM: a terapia deve acompanhar a vulnerabilidade biológica e clínica, e não apenas rótulos de FE ou percepções de recuperação^9,37,38^.

### Biomarcadores, assinaturas metabólicas e plausibilidade biológica

Análises mecanísticas e de biomarcadores avançaram hipóteses sobre os benefícios dos iSGLT2. O programa de ECR EMPEROR avaliou as vias de eritropoiese e mobilização de ferro com empagliflozina na IC, apoiando uma ligação plausível entre a inibição de SGLT2 e a melhora na entrega de substrato hematológico, o que pode influenciar sintomas e *status* funcional^39^. Análises exploratórias em populações com DRC investigaram a redução do ácido úrico como um fenótipo metabólico potencialmente relevante para o risco vascular e renal^40^, enquanto dados de IC aguda examinaram as trajetórias do ácido úrico durante a recompensação^41^. Análises *post hoc* avaliaram ainda mais sinais do metabolismo do ferro em ensaios de iSGLT2 na DRC^42^. Estudos focados em biomarcadores avaliaram os efeitos da empagliflozina em pacientes com IC e DM tipo 2^37^, e biomarcadores relacionados à fibrose, incluindo a galectina-3^43^, em populações com DM tratadas com dapagliflozina^44^. Trabalhos clínicos mecanísticos em DM exploraram marcadores celulares e relacionados a podócitos que apoiam a plausibilidade renoprotetora^45^, e dados experimentais relacionaram a inibição de SGLT2 ao catabolismo renal de aminoácidos de cadeia ramificada^46^.

Uma evolução importante nesse campo tem sido a disponibilidade crescente de biorrepositórios centralizados vinculados a grandes ECR, permitindo análises mais granulares que vão além das associações transversais entre gravidade da doença e níveis de biomarcadores, incluindo respostas dinâmicas de biomarcadores ao tratamento ativo em comparação ao placebo^28,29,31,39^. Essa mudança permite que os biomarcadores sejam interpretados não apenas como marcadores de risco, mas também como indicadores de engajamento biológico e modulação terapêutica.

Paralelamente, percepções derivadas de biópsias renais orientadas por protocolo em populações bem caracterizadas — como a DRC relacionada ao DM e à hipertensão arterial (HA), bem como a injúria renal aguda (IRA) — enriqueceram ainda mais a compreensão mecanística^46^. Iniciativas como o *Precision Kidney Medicine Project* (https://www.kpmp.org/) exemplificam como dados moleculares, histopatológicos e clínicos integrados podem refinar definições de fenótipos e relacionar assinaturas de biomarcadores a vias de lesão e reparo estrutural.

Em conjunto, essas vertentes mecanísticas fortalecem a plausibilidade biológica de benefício multiorgânico e ajudam a interpretar os “efeitos de classe” como modulação convergente de vias descendentes de inflamação, metabolismo e remodelamento tecidual^39,40,41,42,43,44,45,46,47^.

### Prevenção cardiometabólica pós-infarto do miocárdio

Subanálises do DAPA-MI examinaram desfechos cardiometabólicos após IM agudo, de acordo com o *status* glicêmico basal, o índice de massa corporal (IMC) e a FE, alinhando-se a um paradigma preventivo de SCRM que enfatiza a intervenção ao longo de trajetórias de alto risco, em vez de aguardar a IC estabelecida ou a DRC progressiva^48,49^. Trabalhos de enquadramento mecanístico que relacionam a inibição precoce de SGLT2 pós-IM a vias inflamatórias e de senescência reforçam ainda mais o posicionamento preventivo^27^. Em conjunto, a base de evidências de 2025 continua a deslocar os iSGLT2 de uma “terapia posterior para IC/DRC” para uma modificação mais precoce da trajetória de SCRM em contextos selecionados de alto risco^27,48,49^.

### Evidências contextuais em doença sistêmica aguda

Análises pré-especificadas do DARE-19 avaliaram a dapagliflozina em pacientes hospitalizados com COVID-19, com e sem DM tipo 2, acrescentando dados de segurança e desfechos específicos ao contexto para os iSGLT2 em cenários de doença aguda^50^. Esses dados reforçam a importância de definir não apenas quem se beneficia, mas também quando e sob quais restrições fisiológicas agudas a terapia pode ser iniciada e mantida com segurança^50^.

### Consolidação das evidências sobre antagonistas não esteroidais do receptor mineralocorticoide (ARMne) nos fenótipos de SCRM

Em 2025, os ARMne emergiram como uma terapia central na SCRM, particularmente por meio do estudo FINEARTS-HF e de suas análises pré-especificadas, que abordaram desfechos renais, heterogeneidade fenotípica e a biologia de eletrólitos relevante para a segurança.

Uma contribuição importante foi a avaliação dos desfechos renais no FINEARTS-HF, abordando um domínio de evidências historicamente incerto — desfechos renais em populações de IC com graus variados de DRC e carga de DM^10^. Análises pré-especificadas esclareceram como os declínios precoces da TFGe com finerenona devem ser interpretados na prática clínica, enquadrando a trajetória renal inicial como parte do perfil farmacodinâmico esperado, e não necessariamente como um sinal de dano^51^. Isso tem relevância clínica: apoia a continuidade da terapia em pacientes apropriados quando as alterações precoces da TFGe ocorrem em padrões previsíveis e são contextualizadas dentro de compensações de risco-benefício de longo prazo^10,51^.

### Eficácia ao longo da FE e em subgrupos clinicamente relevantes

Análises pré-especificadas avaliaram a finerenona ao longo do espectro de FE^52^, em pacientes com eventos recentes de piora da IC^53^ e entre indivíduos com FE melhorada^54^. Análises adicionais de subgrupos abordaram fragilidade^55^, obesidade^56^, fibrilação atrial^16^ e diferenças relacionadas ao sexo no efeito do tratamento^17^. Os desfechos centrados no paciente foram avaliados por meio de instrumentos de avaliação do *status* de saúde específicos para a doença, incluindo análises do KCCQ (*Kansas City Cardiomyopathy Questionnaire*)^18^, e a estimativa de benefício em longo prazo foi explorada por meio de abordagens de modelagem pré-especificadas^19^. A contextualização por modelo de risco utilizando o PREDICT-HFpEF apoia ainda mais uma abordagem informada por fenótipo para o uso do ARMne^20^. Além da IC, dados de ensaios mecanísticos examinaram a rigidez arterial e biomarcadores cardiorrenais em DM tipo 2 com DRC^57^, reforçando a plausibilidade vascular e renal. Em conjunto, o estudo FINEARTS-HF e suas análises fortalecem a posição da finerenona como uma terapia relevante na SCRM por fenótipo, cujo valor se estende além de um único resultado principal de ensaio, para benefícios interpretáveis, centrados no paciente e clinicamente estratificados^16,17,18,20,53,54,55,56,57,58,59,60^.

### Segurança, biologia do potássio e estratégias para viabilizar a terapia

O manejo de eletrólitos permaneceu clinicamente central. Uma análise FIDELITY avaliou os padrões de hipopotassemia e os desfechos em pacientes com DM tipo 2 e DRC tratados com finerenona^61^. Paralelamente, estudos de manejo de IC examinaram estratégias para viabilizar a terapia otimizada com ARM entre pacientes propensos à hiperpotassemia; o ciclossilicato de zircônio sódico foi estudado como abordagem para facilitar a otimização da espironolactona^62^. As populações em diálise foram abordadas por um grande ECR de espironolactona em diálise de manutenção, informando o papel matizado do ARM esteroidal na DRC avançada^63^. Análises adicionais exploraram padrões de interação entre aldosterona e ARM no EPHESUS^64^, e trabalhos farmacocinéticos examinaram a balcinrenona em insuficiência renal grave^11^, complementando o panorama mais amplo dos ARM relevantes para as populações com SCRM. Em conjunto, esses dados enfatizam que a “implementação do ARM” é cada vez mais uma ciência de viabilização — estratificação de risco, monitoramento e estratégias de mitigação —, e não uma decisão binária de tratar ou não tratar^11,14,62 63,64^.

### Terapia combinada: finerenona e inibição de SGLT2

O paradigma da SCRM enfatiza o bloqueio combinado de vias para reduzir o risco residual. O relatório basal do ensaio CONFIDENCE caracterizou os participantes e um desfecho focado em albuminúria^65^, e uma análise subsequente avaliou o início simultâneo de finerenona e empagliflozina em diferentes estratos de risco renal^66^. Na IC, análises avaliaram os efeitos da finerenona com e sem o uso concomitante de iSGLT2^19^. Um ensaio de estratégia dedicado testou a inibição de SGLT2 com e sem antagonismo da aldosterona na ICFEp (SOGALDI-PEF), interrogando diretamente a estratégia combinada dentro de uma classe sindrômica rica em fenótipos^67^. O MIRO-CKD descreveu o desenho do estudo e as características basais, avaliando balcinrenona e dapagliflozina na DRC^68^, e análises adicionais de subgrupos de DRC abordaram a eficácia e a segurança da finerenona em diferentes estágios de função renal em pacientes asiáticos^69^. Em conjunto, 2025 marca uma transição do entusiasmo conceitual sobre a terapia combinada na SCRM para uma avaliação sistemática — abrangendo desfechos, *endpoints* mecanísticos e estratégias práticas de início^3,4,19,65,66,67,68,69^.

### Terapia baseada em incretinas e fenótipos de SCRM impulsionados pela obesidade

A obesidade e a disglicemia continuam sendo os principais impulsionadores da expressão da SCRM, particularmente na ICFEp. A análise do ensaio SUMMIT examinou como a DRC modifica os efeitos da tirzepatida em pacientes com ICFEp e obesidade — um fenótipo cada vez mais comum na prática contemporânea, em que adiposidade, vulnerabilidade renal e congestão coexistem^12^. Paralelamente, análises de desfechos cardiovasculares dos agonistas do receptor de peptídeo semelhante ao glucagon tipo 1 (aGLP-1) continuaram a explorar a heterogeneidade por FE, utilizando análises derivadas do EXSCEL^14^. Estudos mecanísticos investigaram a captação miocárdica de glicose e marcadores de disfunção diastólica durante a coinfusão de GLP-1/glucagon em adultos com DM tipo 2^15^. Estudos clínicos adicionais avaliaram os efeitos da dulaglutida isolada vs. os da dulaglutida associada a probióticos sobre fatores de risco cardiometabólico^13^, e análises pré-especificadas do ensaio SOUL examinaram os desfechos cardiovasculares da semaglutida oral, estratificados pelo uso basal de iSGLT2^8^.

Mais recentemente, a tirzepatida demonstrou segurança cardiovascular, mostrando-se não inferior à dulaglutida para eventos cardiovasculares adversos maiores (MACE), ao mesmo tempo em que manteve seus efeitos robustos sobre o controle glicêmico e o peso corporal. Embora tenha sido observada uma redução numérica nos eventos, a superioridade não foi alcançada, posicionando a tirzepatida como uma terapia metabolicamente potente, com segurança cardiovascular confirmada, reforçando seu papel no cenário terapêutico em evolução da SCRM^70^.

Novas percepções mecanísticas também surgem do ensaio REMODEL, que destaca como a modulação do receptor de incretina impacta vias de sinalização miocárdica e do tecido adiposo relevantes para o remodelamento, o metabolismo e a inflamação sistêmica nos fenótipos de SCRM impulsionados pela obesidade^71^.

Em conjunto, 2025 apoia uma narrativa integrada de SCRM, na qual as estratégias baseadas em incretinas passam cada vez mais a coexistir com a inibição de SGLT2 e a terapia com ARM — particularmente em fenótipos de IC associados à obesidade, nos quais a trajetória de peso, a biologia da congestão e a limitação funcional se entrelaçam^12,52^.

### Análogos da amilina e o *continuum* cardiovascular-renal-metabólico

Os análogos da amilina são peptídeos sintéticos que mimetizam as ações da amilina, um hormônio cossecretado com a insulina pelas células β pancreáticas em resposta à ingestão alimentar. A amilina atua principalmente no sistema nervoso central, particularmente na área postrema, promovendo saciedade, reduzindo o apetite, retardando o esvaziamento gástrico e suprimindo a secreção pós-prandial de glucagon, contribuindo assim para o controle glicêmico e a regulação do peso corporal. A cagrilintida é um análogo de amilina de ação prolongada em investigação para o manejo da obesidade, com potenciais benefícios nas vias cardiovascular, renal e metabólica. Estudos de fase 2 demonstraram que a cagrilintida induz perda de peso clinicamente relevante (até 10,8% em 26 semanas) e melhora parâmetros metabólicos, com um perfil de segurança comparável ao da liraglutida^72^. Esses achados foram ampliados nos ensaios de fase 3a REDEFINE 1 e 2, nos quais a combinação de dose fixa de semaglutida/cagrilintida (CagriSema) produziu perda de peso substancial e reduções clinicamente relevantes na pressão arterial sistólica e diastólica e no perfil lipídico, apoiando seu papel na redução integrada do risco cardiovascular-renal-metabólico^73,74^.

### Lipídeos, aterosclerose e risco vascular na SCRM

A doença aterosclerótica continua sendo uma contribuinte principal para os desfechos de SCRM. O ECR PURSUIT avaliou um inibidor oral de PCSK9 para a hipercolesterolemia, abordando barreiras de adesão e acesso inerentes às terapias injetáveis e alinhando-se às prioridades de prevenção vascular voltadas à implementação^75^. Análises adicionais do ODYSSEY OUTCOMES focaram em fenótipos de dislipidemia de alto risco, incluindo hipercolesterolemia familiar provável ou hiperlipoproteinemia tipo III^76^. A lipoproteína(a) foi avaliada como marcador de risco vascular na isquemia crônica ameaçadora de membro após revascularização, com atenção a eventos adversos maiores de membro e mortalidade^77^. Além dos lipídeos, um ensaio de fase 2 direcionado à inibição de LOX-1 em pacientes com inflamação residual após IM sinalizou interesse contínuo em estratégias vasculares anti-inflamatórias potencialmente relevantes para populações de SCRM com ativação inflamatória persistente^78^.

Mais recentemente, o ensaio ZEUS ampliou esse paradigma ao direcionar diretamente a sinalização da interleucina-6 com ziltivekimabe em pacientes com DRC e doença cardiovascular aterosclerótica estabelecida, demonstrando reduções significativas em biomarcadores inflamatórios e fornecendo prova de conceito para a modificação do risco vascular direcionada a citocinas em populações com SCRM caracterizadas por carga inflamatória elevada (NCT05021835).

Esses dados vasculares reforçam um princípio central da SCRM: a modificação integrada do risco deve abranger tanto as vias hemodinâmica/renal quanto a biologia aterosclerótica-inflamatória, particularmente em pacientes com vulnerabilidade cardiorrenal sobreposta e risco vascular residual^75,76,77,78^.

### Deficiência de ferro, anemia e o eixo rim-coração

A deficiência de ferro e a anemia continuam sendo contribuintes proeminentes e potencialmente modificáveis para sintomas, *status* funcional e risco de hospitalização na IC e na DRC^79^. O ECR FAIR-HF2 DZHK05 abordou a incerteza quanto à carboximaltose férrica intravenosa na IC com deficiência de ferro^80^, e análises de características basais contextualizaram a população incluída em relação a ensaios prévios sobre ferro^81^. O IRONMAN forneceu novas percepções sobre o risco de infecção com a reposição de ferro^82^ e sobre os efeitos estratificados por idade da derisomaltose férrica intravenosa^83^. Na anemia da DRC, um estudo piloto randomizado avaliou o daprodustate na IC com anemia associada à DRC e examinou as relações entre a resposta da hemoglobina e marcadores do metabolismo do ferro, como a hepcidina^84^. Em conjunto, esses estudos destacam que a agenda da SCRM inclui cada vez mais fatores hematológicos e metabólicos coadjuvantes da congestão, da intolerância ao exercício e da instabilidade clínica — domínios que influenciam de forma significativa os desfechos e a qualidade de vida, mas que frequentemente permanecem subtratados no cuidado cardiorrenal convencional^39,80,81,82,83,84^.

### Congestão, hemodinâmica e injúria renal aguda nos diferentes contextos de SCRM

A tensão entre compensar de forma eficaz um quadro de congestão e preservar a função renal permanece central nos cenários de IC aguda e perioperatórios. O racional do estudo DECONGEST descreveu uma estratégia de terapia diurética guiada por avaliações seriadas de sódio em amostra urinária pontual na IC aguda, com o objetivo de melhorar a eficiência e a segurança do tratamento da congestão^25^. As terapias direcionadas à via da relaxina continuaram a ser estudadas na piora da IC com FE preservada: um ensaio de fase 2 com volenrelaxina examinou a função atrial esquerda, a congestão e a função renal^23^, enquanto os resultados do subestudo de biomarcadores do RELAX-AHF-2 exploraram sinais de proteção de órgãos-alvo^85^. Trabalhos em fase inicial avaliaram os efeitos na perfusão renal de um agonista do receptor de relaxina na IC com FE reduzida (ICFEr)^86^, e um estudo mecanístico de combinação examinou o AZD5462 associado à dapagliflozina na IC com comprometimento renal moderado^87^.

A prevenção e a recuperação da IRA foram investigadas em cenários de cirurgia cardíaca com o ANG-3777^88^ e em ensaios que avaliaram estratégias anestésicas e desfechos de função renal^89,90^. Em conjunto, esses estudos refletem a maturação contínua de uma abordagem da SCRM para a doença aguda: congestão, perfusão, inflamação e lesão renal são cada vez mais examinadas como processos interconectados e modificáveis, e não como dano colateral inevitável do tratamento da congestão ou de procedimentos complexos^24,25,77^.

Apesar da maturação da estrutura da SCRM, os pacientes em diálise de manutenção permanecem sistematicamente sub-representados nos principais ensaios cardiovasculares, renais e metabólicos^1,2,63^. Essa exclusão persistente reflete uma convergência de preocupações metodológicas, regulatórias e biológicas, incluindo riscos concorrentes aumentados, farmacocinética alterada, receios de segurança relacionados à instabilidade eletrolítica e à hipotensão e incerteza quanto aos desfechos apropriados em uma população na qual mortalidade, hospitalização e qualidade de vida frequentemente divergem das trajetórias mais precoces da SCRM^63^. Como resultado, as populações em diálise têm sido frequentemente tratadas como uma condição de fronteira, e não como um *continuum* dentro da biologia da SCRM, reforçando uma lacuna de evidências clínicas desproporcional à sua carga absoluta de risco cardiovascular e metabólico^1,2^.

### Estratificação de risco: de biomarcadores a fragilidade, adiposidade e aprendizado de máquina

A predição de risco e a seleção de pacientes continuaram a migrar de uma classificação de gravidade em eixo único para estruturas integradas baseadas em fenótipo e trajetória. Um modelo validado de aprendizado de máquina multivariável foi desenvolvido para prever o risco cardiorrenal na doença renal diabética (DRD), usando variáveis clínicas e biomarcadores, ilustrando o papel crescente de ferramentas orientadas por dados para guiar a intensificação e a terapia combinada^91^. Em populações com IC, escores nutricionais foram avaliados quanto ao valor prognóstico^92^, e alterações temporais em biomarcadores, função e qualidade de vida foram mapeadas antes dos desfechos adversos na IC com FE levemente reduzida ou preservada (ICFElr/ICFEp)^93^. Medidas de atividade física derivadas de acelerômetros vestíveis foram estudadas como possíveis biomarcadores digitais em populações de ECR de IC^52^. É importante destacar que antropometria relacionada à adiposidade e métricas de obesidade foram avaliadas em análises combinadas de ICFEp/ICFElr com desfechos renais e clínicos, apoiando uma caracterização mais granular da adiposidade além do IMC isoladamente^94,95^. Trabalhos complementares em DM avaliaram marcadores inflamatórios e medidas de neuropatia autonômica cardiovascular, enfatizando que os domínios inflamatório e neuroautonômico podem refinar ainda mais a fenotipagem em populações disglicêmicas de SCRM^96^. Em conjunto, esses estudos apoiam uma definição ampliada de fenótipo de SCRM, incorporando *status* funcional, fragilidade, distribuição da adiposidade, trajetórias de biomarcadores e fenótipos digitais — dimensões que provavelmente melhoram o alinhamento entre o risco biológico e a estratégia terapêutica^4,91,94^.

### Intervenções no estilo de vida e na dieta: emergentes, mas ainda incipientes

As intervenções no estilo de vida receberam atenção direcionada, frequentemente incorporando tecnologia digital e perfilamento metabolômico. Um ensaio piloto randomizado comparou uma dieta cetogênica associada a suporte digital com uma dieta de baixo teor de gordura, avaliando seus efeitos sobre a saúde cardiorrenal e metabólica em uma população predominantemente hispânica com sobrepeso ou obesidade, por meio de análises metabolômicas e ferramentas de saúde digital^96^. Outros estudos investigaram intervenções dietéticas e baseadas em suplementos, incluindo o uso de miméticos do glicocálix em indivíduos com DM tipo 2, com avaliação por classificadores peptidômicos urinários^97^. Além disso, estudos exploratórios de fisiologia examinaram estratégias para atenuar biomarcadores associados à IRA durante a exposição ao calor^35^. Evidências recentes também têm chamado atenção para o papel prejudicial dos alimentos ultraprocessados (AUP) na SCRM. Além de seu alto teor de carboidratos refinados, gorduras não saudáveis, sódio e aditivos alimentares, os AUP têm sido consistentemente associados à inflamação sistêmica, ao estresse oxidativo, à resistência à insulina, à dislipidemia, à disfunção imunológica e a alterações na microbiota intestinal, mecanismos que, em conjunto, promovem a progressão da doença cardiovascular, renal e metabólica^98^. Esses achados reforçam que as recomendações dietéticas na SCRM devem enfatizar não apenas a restrição calórica e a redução de peso, mas também melhorias na qualidade geral da dieta, priorizando padrões alimentares minimamente processados e limitando o consumo de AUP. Embora cientificamente informativos, esses estudos permanecem, em grande parte, geradores de hipóteses em relação aos ensaios de desfecho farmacoterapêutico, ressaltando a necessidade de avaliações maiores, adequadamente dimensionadas e orientadas por desfechos, das vias de cuidado na SCRM baseadas no estilo de vida^97,99,100^.

Os principais avanços revisados ao longo de 2025 convergem para um modelo integrado da SCRM, no qual a compreensão da biologia compartilhada, o uso de terapias modificadoras da doença e a implementação orientada pelo fenótipo redefinem a abordagem contemporânea do cuidado cardiovascular-renal-metabólico (Figura 2).

**Figura 2:** Manejo contemporâneo da síndrome cardiovascular-renal-metabólica (SCRM): terapias modificadoras da doença e implicações clínicas.

### Implicações para a prática clínica e prioridades de pesquisa

Em 2025, o paradigma da SCRM avançou ainda mais do conceito para a estratégia operacional. Primeiro, as evidências apoiaram a implementação mais precoce e consistente de terapias fundamentais, incluindo os iSGLT2 iniciados durante a hospitalização por IC, com dados consistentes com a prevenção cardiometabólica logo após o IM^36,48,87^. Segundo, a base de evidências apoiou cada vez mais a terapia combinada racional para reduzir o risco residual, particularmente por meio da expansão dos dados da finerenona nos desfechos de IC e renais e por meio de ECR e análises que avaliaram ou contextualizaram o uso concomitante com iSGLT2^19,65,66,67^. Terceiro, o cuidado gravitou cada vez mais em direção a uma interpretação orientada por fenótipo, além dos rótulos de órgão único, à medida que a fragilidade, as distribuições de adiposidade, as trajetórias de biomarcadores, as ferramentas de risco de aprendizado de máquina e os desfechos relatados pelo paciente se tornaram centrais para o direcionamento do tratamento e a modificação do efeito^18,55,91,93,94^. Por fim, uma incerteza importante persiste em cenários de DRC avançada e de cuidado agudo, nos quais as populações em diálise, a IRA perioperatória e as estratégias de tratamento da congestão na IC aguda permanecem áreas ativas, com padrões de prática heterogêneos e evidências incompletas de desfechos^63,88,101^.

Olhando para o futuro, três prioridades emergem: (1) são necessários ensaios definitivos de desfechos de regimes combinados racionais em populações com SCRM cuidadosamente fenotipadas, para avançar além da extrapolação e em direção a uma verdadeira estratificação personalizada de vias^3,4,91^; (2) estudos pragmáticos de implementação devem abordar as lacunas de adoção — momento de início, sequenciamento, persistência, monitoramento e equidade —, pois a magnitude do benefício observada nos ECR não se traduzirá na prática se a terapia for retardada, descontinuada ou implementada de forma inconsistente entre os sistemas^1,3^; (3) a integração segura e equitativa de biomarcadores digitais, ômicas e suporte à decisão por aprendizado de máquina nos fluxos de trabalho do mundo real deve ser priorizada, com atenção cuidadosa ao viés, à validade externa e à responsabilização clínica^4,91^. A implicação geral de 2025 é que o cuidado na SCRM é cada vez mais definido por intervenção mais precoce, estratégias combinadas e aplicação guiada por fenótipo, equilibradas em relação às restrições de implementação e às lacunas de evidências persistentes nos extremos da DRC e da doença aguda^1,3,4,102^.

### Considerações metodológicas e cautela na interpretação

Embora a literatura sobre SCRM de 2025 tenha avançado substancialmente em relação à integração terapêutica e à compreensão mecanística, é importante reconhecer que nem todas as evidências randomizadas produziram resultados inequivocamente positivos. Vários ECR contemporâneos e análises pré-especificadas demonstraram efeitos neutros sobre desfechos duros, como mortalidade por todas as causas ou cardiovascular, apesar de sinais favoráveis em desfechos secundários, biomarcadores ou desfechos relatados pelo paciente^9,63,80,88^. Esses achados ressaltam o desafio persistente do risco residual em estados avançados de SCRM e alertam contra a superinterpretação de desfechos substitutos ou intermediários como evidência definitiva de modificação da doença — particularmente em populações caracterizadas por alto risco concorrente, seguimento curto ou tamanho amostral limitado.

Uma consideração metodológica mais ampla se refere à possível influência do viés de publicação e da fragmentação analítica dentro da crescente base de evidências sobre SCRM. Análises de subgrupos positivas, estudos *post hoc* de biomarcadores e sinais enriquecidos por fenótipo podem alcançar publicação de forma preferencial, enquanto achados neutros ou negativos — especialmente aqueles relacionados ao sequenciamento terapêutico, a estratégias combinadas ou a falhas de implementação — permanecem sub-representados^63,80^. Além disso, a disseminação de análises pré-especificadas e exploratórias em populações de ECR sobrepostas corre o risco de fragmentar a interpretação e inflar a consistência percebida do benefício. Reconhecer essas limitações é essencial para evitar extrapolações prematuras e assegurar que a estrutura da SCRM evolua com humildade científica apropriada, equilibrando o entusiasmo pela integração baseada em vias com uma avaliação rigorosa da incerteza, da heterogeneidade e da validade externa^9,63,80,88^.

## Data Availability

Os conjuntos de dados gerados e/ou analisados durante o presente estudo estão disponíveis com o autor correspondente, mediante solicitação razoável.
